# Conceptual frameworks and empirical approaches used to assess the impact of health research: an overview of reviews

**DOI:** 10.1186/1478-4505-9-26

**Published:** 2011-06-24

**Authors:** Rita Banzi, Lorenzo Moja, Vanna Pistotti, Andrea Facchini, Alessandro Liberati

**Affiliations:** 1 Centro Cochrane Italiano, Istituto Ricerche Farmacologiche Mario Negri, Milano, Italia; 2 Dipartimento di Sanità Pubblica-Microbiologia-Virologia, Università degli Studi, Milano, Italia; 3 Istituto Ortopedico Rizzoli, Bologna, Italia; 4 Università degli Studi di Bologna, Bologna, Italia; 5 Dipartimento di Oncologia, Ematologia e Malattie dell'Apparato Respiratorio, Università di Modena e Reggio Emilia, Modena, Italia; 6 Agenzia Sanitaria e Sociale Regionale dell'Emilia Romagna, Bologna, Italia

**Keywords:** Research governance, Research impact, Health research, Bibliometrics

## Abstract

**Background:**

How to assess the impact of research is of growing interest to funders, policy makers and researchers mainly to understand the value of investments and to increase accountability. Broadly speaking the term "research impact" refers to the contribution of research activities to achieve desired societal outcomes. The aim of this overview is to identify the most common approaches to research impact assessment, categories of impact and their respective indicators.

**Methods:**

We systematically searched the relevant literature (PubMed, The Cochrane Library (1990-2009)) and funding agency websites. We included systematic reviews, theoretical and methodological papers, and empirical case-studies on how to evaluate research impact. We qualitatively summarised the included reports, as well the conceptual frameworks.

**Results:**

We identified twenty-two reports belonging to four systematic reviews and 14 primary studies. These publications reported several theoretical frameworks and methodological approaches (bibliometrics, econometrics, ad hoc case studies). The "payback model" emerged as the most frequently used. Five broad categories of impact were identified: a) advancing knowledge, b) capacity building, c) informing decision-making, d) health benefits, e) broad socio-economic benefits. For each proposed category of impact we summarized a set of indicators whose pros and cons are presented and briefly discussed.

**Conclusions:**

This overview is a comprehensive, yet descriptive, contribution to summarize the conceptual framework and taxonomy of an heterogeneous and evolving area of research. A shared and comprehensive conceptual framework does not seem to be available yet and its single components (epidemiologic, economic, and social) are often valued differently in different models.

## Background

It is widely accepted that research is a crucial investment to foster innovation, knowledge advancement, and social and economic development. For example a knowledge gain is assumed to result from biomedical and basic research; if such an output is then properly translated in a short but reasonable time lag, it will lead to a better health status for populations and patients. Much of the information produced is not easily transferable to patient care and this has led to the concept of the so called "translational blocks" [1]. Evidence produced by applied types of health research, such as the "comparative effectiveness" and "health services research" elicits its potential impact in a more straightforward way. Health care systems, which are nowadays increasingly keen to directly support research, are interested in overcoming the translational blocks and to facilitate a quicker return of their investment in terms of information that would help selecting the more effective and cost effective interventions so that quality and appropriateness can be maximised [2-4].

By definition, research activities are risky and their returns highly unpredictable. So, any attempt to increase the research system's effectiveness, and to assure and monitor quality, is welcome by the whole scientific community and funders [5]. Competition on limited resources and different funding modalities also raise additional concerns. From the limited available evidence on the proportion of investments by research stream, funding is skewed toward biomedical and basic research which, by definition, require more time to have an impact [6]. This has raised a debate between those who ask for a priority-setting based on the ability to produce relevant, usable, and transferable outputs and those supporting the view that research should be driven only by the researchers' interest. If left only into the close boundaries of the "research communities", there is a concrete risk that the priority setting becomes self-referent and the "bidirectional dialogue" between those that generate relevant questions from observation in clinical practice and those that are responsible to generate the new knowledge remains very limited [7]. Monitoring and measuring research impact is a complex objective requiring the involvement of many actors within the research pipeline. In the past two decades, many theoretical frameworks and methodological approaches to measure research impact and returns have been developed. The payback [8], the cost-benefit [9,10], and the decision making impact models [11] are examples of evaluation approaches reported in scientific and health policy literature. A partial list with a qualitative description of the most common frameworks is reported in Table 1. All these models share a multidimensional approach achieved by categorizing impact and benefit in many dimensions and trying to integrate them [12]. A set of indicators and metrics are then generally associated to each category of impact. For example, bibliometric indicators (e.g. impact factor) are highly reported as a measure of the diffusion and awareness of research results. These indicators, though welcome to some extent because of their directness, are at best only surrogate measures of impact. Moving toward more robust metrics, such as those measuring the health status or the economic benefit of a population, is a complex task but in some way essential [13].

**Table 1:** A qualitative description of the most widespread frameworks for the evaluation of research impact

Model	Description	Dimensions of impact evaluation	Main proposed indicators	Examples and Main bibliographic references
1. Payback	Organizes together in a sequential and systematic way the different aspects on the impact of research projects from dissemination to potential benefits for health care	i) knowledge production; ii) research targeting and capacity building; iii) informing policies and product development; iv) health benefit; v) broader economic benefits.	i) journal articles, conference presentations, books, research reports, other disseminative material; ii) new research lines or know how, career promotions, PhD, masters; iii) guidelines and documents addressing policies citations, membership of decision panels; iv) health outcomes, QALY, savings for health care systems v) benefits in occupation and economic development, productivity	Research programs financed by the NHS UK [12,38,40,44]
2. Research Impact	Evaluates the influence of research results and of the potential concurrent/competing factors (cultural context, policy content, decision process) in policy making	i) research related impact ii) policy impact iii) service impact iv) societal impact	i) knowledge and methodology advancements, networking, leadership ii) establishment of collaborations and networks iii) evidence-based practice; cost-containment, quality of care; iv) knowledge, attitudes and behaviour, health literacy, social capital, equity, macroeconomy	London School of Hygiene and Tropical Medicine researchers evaluation [45]
3. Research utilization ladder	Evaluates the ways in which research progresses towards its application by practitioners and policy makers	i) transmission (of research results to practitioners and policy makers) ii) cognition (reading and understanding) iii) reference (quoting of research results in reports, studies, actions) iv) effort (to adopt research results) v) influence (on choices and decisions) vi) application	-	[46]
4. Lavis decision-making impact model	Evaluates the impact on decision making of any individual or organisation, considering the target audience of research and the resources available for the assessment	i) policy makers are the ones seeking research (*user-pull*) ii) researchers actively disseminating results (*producer-pull*) iii) researchers and policy-makers are both involved actively (*exchange measures*)	Process measures (if limited resources are available) Intermediate outcome measures (by performing surveys) Outcome measures (by performing cases studies)	Canada [11]
5. Weiss Logic Model	Analyzes the ratio between input (resources), process (activity) and results of research (products)	i) initial benefits ii) intermediate benefits iii) long-term benefits	Output: publications i) awareness of medical research results in policy making ii) any changes in practice iii) any changes in well-being and health	[47]
6. HTA Organisation assessment framework	Effectiveness is measured by the ability to impact on decision making	i) productivity ii) capacity to attract and maintain resources and to mobilize external support iii) culture and values maintenance (independence in action, transparency of the process, accountability to stakeholders)	i) volume and productivity of outputs, quality, comprehensiveness, and accessibility ii) measure of visibility and credibility	Quebec Council on Health Care Technology, Canada [48]
7. Societal Impact framework	Research is considered as the valuation of the communication of research groups with relevant surroundings (industry, general public, scientific community, public and policy institutions)	i) knowledge products ii) knowledge exchange and esteem iii) knowledge use iv) attractiveness	i) publications, patents, products ii) presentations, consultancies, and public lectures iii) citations, product use iv) further funding	Royal Netherlands Academy of Arts and Sciences 2002 [49,50]
8. Balanced scorecard	Measures performance and drives organizational strategy by incorporating organisational aspects together with financial performance	i) financial; ii) customers; iii) business process; iv) learning and growth.	-	[51] Ontario University Health Network [52]
9. Research Assessment Exercise (RAE)	To produce quality profiles for each submission of research activity made by UK academic institutions	Three quality profiles are defined (panel decides the weight given to each profile): i) research output (minimum 50%); ii) research context (minimum 5%); iii) other indicators (minimum 5%).	i) RAE1: staff information (volume and type of contracts, external collaborators), analysis of funding for research fellows; ii) RAE2: research output (publications, patents, reports, database, software, etc); iii) RAE3: research scholarships iv) RAE4: attractiveness for external funding v) RAE5a: further information on groups of research (networking, development of a research culture, etc)	RAE 2008 [53]
10. Cost-benefit Analysis	Research impact evaluated in monetary terms	i) savings for health care systems (direct costs) ii) savings for the community on the whole (indirect costs)	i) QALY ii) profits	NIH, USA [54] National Institute of Health 1993 [55]

The objective of this overview is to describe the conceptual and methodological approaches to evaluate the impact of biomedical and health research. Specifically, we aimed at collecting and qualitatively summarizing what is available in the biomedical literature in terms of theoretical framework and methodologies, with a specific focus on the most valid and reliable indicators of impact. Our objective was also to see whether this qualitative analysis would have allowed the identification of a preferred model to measure research impact and to identify the desirable elements (i.e. dimensions to be considered, robust and reliable indicators) that a reference model should have.

Other key elements in the "research governance debate" such as the analysis of different modes and approaches to research funding, prioritization of topics, or the analysis of barriers and facilitators to the translation of research results were beyond the scope of this overview.

## Methods

In the context of this overview, the term "research impact" refers to any type of output of research activities which can be considered a "positive return" for the scientific community, health systems, patients, and the society in general. We refer to any type of health-related research, basic and biomedical, such as new drug or technology development - and applied research such as clinical trials, health service research, and health technology assessment (HTA).

The complexity and heterogeneity of the topic made the conceptualization of this overview much less straightforward than typical review on medical interventions. We therefore followed the methodology recommended by the Cochrane Collaboration for preparing "overviews of review" rather than following the steps involved in critically appraising the primary studies for a systematic review (SR) [14]. We first searched for SRs summarizing theoretical model or methodological approaches as well as empirical assessment of health research funding programs. To increase the comprehensiveness of our search we also sought primary studies (case studies) not included in the selected SRs or published in languages other than English. We included studies describing conceptual or methodological approaches to evaluate the impact of health research programs and the empirical evaluation of specific programs, funders, research teams, clinical area, etc. In both cases, to be eligible for this review the study should have mentioned specific impact categories and the indicators and metrics used to measure this impact.

Given the broad perspective of the review, the methodology to identify relevant studies comprised an iterative process. We first performed a systematic search on bibliographic databases (Medline and The Cochrane Library) using a modified version of the search strategy proposed by Martin Buxton and collaborators [12]. The search was limited to the SRs published between 1990 and 2009. We also tried to include relevant studies and reports not included in the eligible SRs (i.e. publications in French, Italian, and Spanish) published between 2007 and 2009. Besides bibliographic databases, we screened the research funding and Charity's Foundations websites cited in the eligible studies, looking for the grey literature (i.e. additional reports not indexed in bibliographic databases). In fact, a large part of the literature in this field would be made up of heterogeneous publications and critical appraisal reports published by the main funding agencies. Details on the search strategies used and the searched websites are reported in Additional file 1.

Finally, we screened the citations reported in the included publications and assessed the literature in the field already retrieved by authors. We did not contact study or report authors.

Two reviewers independently selected relevant publications by screening titles and abstracts. After the retrieval of the selected full text publications and, if needed, of associated publications, we extracted the following details: objective, country and setting, evaluation time lag, conceptual model methodology, main results and conclusions.

From the analysis of the literature and with reference to the more widely accepted theoretical models we attempted a description of the categories of impact more frequently measured, focusing on the indicators and metrics for each category.

Given the heterogeneity of study designs, the different objectives and the lack of a standard methodology between studies, we did not perform a quality assessment of the methods used in different studies.

## Results

**Figure 1:**
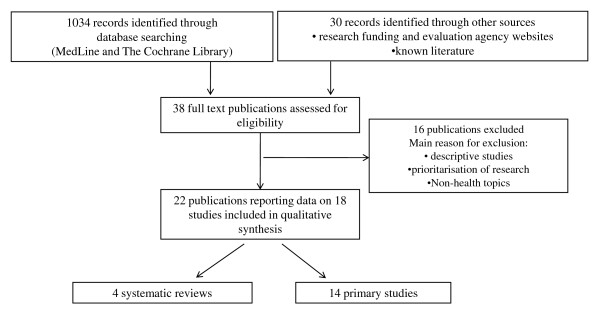
**Flow of studies through the different phases of the overview**.

From the bibliographic databases and web searches we identified 1064 records. Among these 38 potentially relevant publications were retrieved as full text (Figure 1). Sixteen publications were excluded, i.e. because descriptive, [15] or dealing with prioritization of research topics rather than impact [16,17]. We included 22 publications, referring to 18 studies: four SRs [12,18-21] and 14 primary studies [8,9,22-36]. For each study, we synthesized the overall objective, scientific area evaluated, country and setting, and the time lag of the evaluation.

Hereafter we briefly presents the four SRs. For details please refer to Additional file 2. The included SRs presented and discussed comparative analyses of theoretical models and empirical evaluations performed in several countries from 1990. They all used a public funder's viewpoint (e.g. central or regional governments, WHO). Besides the evaluation of specific research programs, each SR also reported a description of theoretical models used as a framework for the assessment and a more or less explicit description of categories of impact and indicators used in the evaluation. Both the reviews by Hanney et al and the one from the Canadian Academy of Health Science answered to the broad question "how to measure research impact". The first aimed at assessing how the impact of the UK National Health Service Health Technology Assessment program should be measured and collected the available models, their strengths and weaknesses [12]. The latter was interested in defining the impact of the Canadian health research and to answer to the broad questions "is there a best method to evaluate the impacts of health research in Canada?" and "are there best metrics that could be used to assess those impacts?" [20]. These SRs included studies aimed at describing conceptual or methodological approaches to evaluate the impact of health research programs as well as empirical applications of different assessment strategies (desk analysis, interview, peer-review evaluation, case-studies, etc.) and tools for measuring impact (indicators and metrics). The review published by Buxton et al in 2004 focused on the estimation of the economic value of research to society. The review reported an analysis of benefits in terms of direct cost savings to the healthcare system, commercial development, healthy workforce, and intrinsic value to society due to the health gain [18]. Lastly, Coryn et al. have reported a comparative analysis of 16 national models and mechanisms used to evaluate research and allocate research funding [21].

Hereafter we briefly presents the results of the included primary studies. For details please refer to Additional file 3. The studies covered a broad range of evaluation exercises sponsored by public and private research funding agencies. All studies have been conducted in UK, Australia, Canada, and USA, with few exceptions [26,32]. These studies were highly heterogeneous in terms of the applied theoretical frameworks and methodology. The unit under evaluation included researchers (from one single researcher to teams and whole institutions) but also medical discipline (e.g. cardiovascular disease research) or type of grants (e.g. from public institutions, charities, foundations, etc.).

The large majority applied a bottom-up evaluation approach, where information goes from any "producers" of research to any target of research outputs [37]. Two studies applied a more strictly econometric approach used to estimate return on investments in a top down manner [9,36]. The method often used were the desk analysis, peer reviewer evaluation, interviews and questionnaires to principal investigators or to stakeholders with a variety of roles in the research production and utilization.

Across the majority of the SRs and primary studies, research impact was assessed alongside several dimensions, which can be grouped into five categories: "advancing knowledge", "capacity building", "informing decision-making", "health benefits", and "broad socio-economic benefits". Each category, further split into subcategories, had a set of indicators and metrics capable of providing the size of the impact (see Table 2). The more frequently quoted dimensions of impact were advancing knowledge (using bibliometric and citational approaches), capacity building (mainly using a desk analysis approach), and informing decision-making (through the evaluation of how and to what extent research findings are included into the decisional processes, i.e. guidelines). The potential benefits of a research activity on population's health or its socio-economic status were more rarely addressed by the literature as they are, obviously, less directly linked and more complex to assess. In other words, these categories, with their respective indicators, span into a gradient going from surrogate but easy to be measured outcomes, (i.e. bibliometric and citational data) to demanding but relevant outcomes (i.e. morbidity, quality of life). Bibliometric indicators (number of publications, impact factor, citation indexes, etc.) were a case in point here. They were widely considered, reported, and to some extent, accepted due to the fact that they are easy to measure and outputs that can be straightforwardly attributed to a specific research activity. Only the studies adopting an econometric viewpoint [18] or evaluating a specific research area, such as primary care [27,28] or health system effectiveness [29] did not quote (or quoted with less emphasis) bibliometric indicators.

**Table 2:** Description of possible impact categories and relative indicators (*adapted from Canadian Academy of Health Science *[20])

Impact category	Proposed Indicators	Data collection methodology	Level of application	Theoretical Models quoting this category	Advantages	Disadvantages
Advancing knowledge	• Activity (number of peer-reviewed publications absolute or relative - e.g. to the department publications); • Quality (impact factor, relative citation impact, high impact publications, download numbers); • Outreach (co-author analysis, field analysis of citations); • Context and structure (relative activity index); • Other possible indicators (expanded relative citation impact, relative download rate).	Bibliometric and citational analysis; desk analysis.	Basic, clinical, applied and social research. Assessment of individual researchers, teams, institution, projects, funding agencies.	Payback, research impact, research utilization ladder, Lavis decision-making impact model, societal impact, RAE	-wide range of applications -close to research itself -objective -attributed relatively straightforwardly -accessibility and feasibility -limited cost	-surrogate indicators; -not always relevant; -not comparable across different disciplines; -robust only if based on a sufficient set of publications (not fully appropriate to individual researcher evaluation).
Capacity building	• Staff (number of PhD, Master, researchers, member of staff); • Funding (external sources of funding); • Infrastructure (grant for infrastructure and coordination activities); • Other possible indicators (receptor and absorptive capacity)	Desk analysis, database and interviews	Basic, clinical, applied and social research Assessment of teams, institution, projects, funding agencies. Not recommended at individual level.	Payback, research impact, research utilization ladder, Lavis decision-making model societal impact, RAE	-wide range of applications -quite close to research itself -attributed relatively straightforwardly (partially) -accessibility and feasibility -limited cost	--surrogate indicators; -quite subjective; -attribution issues; -self referenced; -non mutually exclusive (*double counting*)
Informing policies and product development	• Health care (guidelines and policy documents citations - e.g. regional plans, educational material, panel representatives) • Research (references used as background for successful funding proposals, consulting and support activity, curricula citations) • Industrial (patents and industrial collaboration, clustering) • Citizens (informative packages, dissemination activities) • Media (journals, radio, tv, web)	Desk analysis, database and interviews	Clinical, applied, social research Assessment of individual researchers, teams, institution, projects, funding agencies.	Payback, research impact, research utilization ladder, Lavis decision-making model, societal impact	-Optimal for projects funded ad hoc to inform decision making -robustness -relevance -feasibility -limited cost	-limited spectrum of application -time-lag between input (research) and output (result) --quite subjective -self referenced
Health and health sector benefits	• Health (Epidemiologic data, incidence, prevalence, mortality QALYs^1^, PROMs^2^) • Health determinants (risk factors, educational and social level of cohesion, pollution) • System (patient satisfaction, waiting lists, compliance and adherence to clinical guidelines, hospitalization, length of inpatient stay, adverse effects/complications)	Desk analysis, database and interviews Case studies, audit	Clinical and applied research Evaluation of teams, institutions, projects, funding agencies	Payback, research impact, research utilization ladder	-robustness -relevance	-many confounders -feasibility and cost of data collection; -time-lag between input (research) and output (result) -possible underestimation of real impact -attribution issue
Economic and social benefits	• Economic rent (salaries, employments) • Licensing returns • Product sales revenues • Spin-off companies • Health benefit (QALY and PROM per health care dollar) • Well-being (happiness, level of social isolation) • Social benefits (socio-economic social)	Desk analysis, database and interviews Case studies, audit Econometrics	Clinical and applied research Evaluation of teams, institutions, projects, funding agencies	Payback, societal impact, cost-benefit.	-robustness -relevance	-many confounders -feasibility and cost of data collection; -time-lag between input (research) and output (result) -possible underestimation of real impact -attribution issue -use of models and assumptions

## Discussion

### Main findings

This overview of reviews shows that the assessment of the impact of, or benefits from, health research is an issue of growing interest, mainly in those countries (UK, Canada, Australia, USA) that invest more in research. Research in this area focuses on three broad areas: i) theoretical frameworks and models aiming at assessing research impact with respect to multidimensional and integrated categories; ii) methodological approaches to the evaluation exercise; and iii) development of valid and reliable indicators and metrics.

A common and key feature of most of the used models is the multidimensional conceptualization and categorization of research impact. Different impact aspects are connected and integrated using a variety of theoretical approaches (i.e. Logic model for the Payback framework). Assessment of research impact that consider more than one category are indeed valued for their ability to capture multifaceted processes.

Several empirical approaches have been used to practically assess research impact: desk analysis, including bibliometrics, peer reviews, interviews, *ad hoc *case studies. The latter seems a reliable methodology: case study implies an explicit and a priori choice to start and conduct an evaluation exercise with specific aims and features. However, they can be at risk of "conceptualization bias" and "reporting bias" especially when they are designed or carried out retrospectively. Finally, feasibility and costs of case studies are also a major barrier to their conduct and subsequent use. In general, the methodology should be as flexible and adaptable as possible to many assessment questions, viewpoints, settings, and type of research and should guarantee the quality of collected data.

The lack of standard terminology, the multifaceted nature of the evaluation, and the heterogeneity of the empirical experiences make it hard to identify a preferred model. The most cited impact dimensions are related to knowledge, public health and socio-economic advancements. The Payback model, [38-41] and its adaptation into the Canadian framework [20] emerged as the most frequently quoted. Both are based on explicit assumptions (positive and negative), have been applied to empirical evaluation, and produce transparent categories of impact, indicators, and limitations of the models. They can be considered comprehensive as assure a global approach to the evaluation of biomedical and health research impact. The identification of appropriate indicators is a critical step in any impact assessment exercise, and assessing research impact makes no exception to this. Indicators can be defined as factors or variables that provide simple and reliable means to measure impact, changes to an intervention, or performance [42]. Ideally, a set of a few robust, valid, shared, transferable, comparable, and feasible indicators able to synthesize research impact should be developed for any assessment. As a matter of fact, the usefulness of indicators highly depends on the evaluation purposes and the level of aggregation of the unit of analysis: for instance, citation indicators partially capture the impact on knowledge advancements as they only consider published literature, they are artificially skewed by journal's impact factor and can be misleading when applied to individuals. Moreover, an indicator itself informs only on a single aspect of research impact, thus sets of indicators are always advisable.

This overview highlights the methodological limitations of the studies carried out in this field, which are briefly summarized below.

First, the vast majority of the studies were retrospective, based on interviews to principal investigators or funders, and mainly focused to record the projects' achievements rather that their pitfalls and limitations. This could lead to several biases, such as selective recall or reporting of (positive) results. The second major limitation is linked to the "attribution", the possibility to postulate a causal link between observed (or expected to be observed) changes and a specific research activity [19]. Another limitation is linked to the possibility of understanding what would have happened without "that" research activity (counterfactual). As very rarely a "control" situation is available, the identification of baseline measures and context factors is important in understanding what any counterfactual might have looked like. Finally, it is not commonly appreciated that substantial time lags exist between research funding and measurement of outputs. An impact assessment should be planned choosing an appropriate time window, which highly depends on the considered type of research and dimension of the impact.

### Limitations of this overview

The main limitations of this overview concern the study retrieval process and the definition of the eligibility criteria. We experienced several difficulties in planning the search strategy, all caused by the heterogeneity of definition and the lack of a standard terminology to describe "research impact". Bearing this in mind, we adopted an approach capable of maximizing sensitivity rather than specificity, that is the application of broad inclusion criteria and the use of several sources of information, not only bibliographic databases. As expected, only 30% of the included publications were found through the traditional biomedical databases (i.e. Medline). Beyond a high variability in the way of indexing these articles, this could be due to a limited interest in the publication of these evaluations, which were considered, at least in the last decade, an administrative duty rather than a scientific activity. Thus many relevant studies were retrieved in the "grey literature" (i.e. funding agency's reports) and not in scientific journals. Even if we were not able to apply a systematic approach to website consultation, we believe this effort had increased the comprehensiveness of our search.

Another methodological limitation of this overview is that we did not estimate the level of publication bias and selective publication in this field. Finally, as our analysis include study up to 2009, we did not capture new important emerging approaches to impact assessment, such as the Research Excellence Framework (formerly RAE) [43].

## Conclusions

The main message of this overview is that the evaluation of the research impact is as yet a heterogeneous, and evolving discipline. Multidimensional conceptual frameworks appear to be adequate as they take into account several aspects of impact and use advanced and analytical approaches (i.e. epidemiologic, economic, and social) to their evaluation. It remains to be clarified how different impact dimensions should be valued and balanced by assessors to fit them to their specific purposes and contexts. Added values to the multidimensional approach are to pursue an explicit planning of the assessment exercise and to carried out the alongside the development of research programs, through monitoring and prospective data collection.

This overview should be seen as a preliminary step toward a shared conceptual framework and taxonomy to assess research impact rather than an indication of an ultimate model, that probably appears unrealistic.

## Competing interests

The authors declare that they have no competing interests.

## Authors' contributions

All authors were involved in the conception of the study. RB, LM, VP, and AL were responsible for the study design and acquisition, analysis, and interpretation of data. VP was responsible for the search strategy design and the study retrieval. All authors contributed to the preparation of the final text of the article, they critically revised it for important intellectual content and gave final approval to the manuscript.

## Source of funding

This overview was developed within the project "Research impact in Emilia Romagna: an open archive of health research outputs. Evaluation of research products and capacity building" funded by the Programma di Ricerca Regione Università 2007-2009, Regione Emilia Romagna, Italy.

The funder had no role in study design, collection, analysis, interpretation of data, writing of the manuscript, and in the decision to submit the manuscript for publication.

We acknowledge that this paper is a revised version of a first overview published in Italian in the journal "Politiche Sanitarie" (http://www.politichesanitarie.it, Banzi, R. Pistotti, V., Moja, L., Facchini A., Liberati A. Valutazione dell'impatto della ricerca biomedica e sanitaria: revisione sistematica di letteratura Politiche Sanitarie 2010, 11(3): 175-195).

## Supplementary Material

Additional file 1 **Appendix 1**. Search strategy used for Medline (up to May 2009) and website searched to retrieve relevant report not published in the scientific journals Click here for file

Additional file 2 **Table s1**. Qualitative description of the included SRs Click here for file

Additional file 3 **Table s2**. Qualitative description of primary studies not included in the previous mentioned reviews Click here for file
