# Supplementary material for: Conceptual frameworks and empirical approaches used to assess the impact of health research: an overview of reviews
Source: Health Res Policy Syst. 2011 Jun 24;9:26. doi: 10.1186/1478-4505-9-26 (PMC3141787; doi:10.1186/1478-4505-9-26)
Supplement: Additional file 1 — Appendix 1. Search strategy used for Medline (up to May 2009) and website searched to retrieve relevant report not published in the scientific journals [file 1478-4505-9-26-S1.DOC]

**Additional file 3, Table S3. Qualitative description of primary studies not included in the previous mentioned reviews**

| **Reference** | **Aim and Focus** | **Country** | **Time lag** | **Commissioner** | **Methods** | **Main Results** | **Main conclusions** |
| --- | --- | --- | --- | --- | --- | --- | --- |
| Hanney 2007, Raftery 2009 [12,22] | To assess the impact of the English NHS  Health Technology Assessment (HTA) program | UK | 1993-2003 | Public funder: NHS | Methodological approach:  -payback framework  - survey of lead investigators and case studies (interviews, evaluation of administrative documents, bibliometric analysis, citation analysis-specifically with NICE guidelines and other NHS documents)  Type of projects analysed:  -projects on primary research (mainly randomized controlled trials): 9 case-studies detailed  -secondary research, (including SRs, meta-analysis and modelling of cost-effectiveness): 4 case-studies detailed  -NICE Technology Assessment Reports (TAR): 3 case-studies detailed | Survey response rate : 65%  Mean number of peer-reviewed publications per project: 2.93 (3.82 for the primary research; 3.36 secondary; 1.81 TAR).  A total number of presentations per project: 5.2 (55% to academic audiences, followed by those  to practitioners and relatively few to service users).  46% of the projects went on to receive further funding.  75% of the projects claimed to have had an impact on policy and 42% on behaviour.  A correlation between high impact projects and the value of the relative publications was found.  The case studies confirmed the survey findings. | HTA programs have a relevant impact on the number of publications, health care policies and behaviour.  It is not possible to study what would have changed without the specific research program (counter-factual ) |
| Hanney 2006 [23] | To assess the impact of a diabetes research | UK | 1980-2001 | Public funder: National Health Service Executive | Case studies (focus on a group of researchers).  Methodological approach:  -standard bibliographic analysis;  -categorization of publication and citations;  -qualitative assessment using surveys, critical pathway analysis by, and interviews  of, co-authors and external experts. | Publications:  1st generation: 29  2nd generation: 799  3rd generation: 12,891  Average citations 1st generation: 27  Survival time 1st generation: 2-11 years  Categorising of citations: 9% classified in the “important and fundamental/basic category”  Qualitative analysis:  25/74 of the team leader co-authors completed the critical pathway data and the questionnaires;  29 paper analysed;  report of strong basis for subsequent career development;  moving to commercial sector | Multidimensional approach essential. The main difficultly is to attribute a specific progress or results. |
| Wooding 2005 [8] | to systematically review and document the outputs and outcomes of  research funded by the Arthritis Research Campaign (ARC) | UK | 1990-1994 | Charity: ARC | Methodological approach: payback framework  Case studies (bibliometric analysis, literature and archival document review and key informant interviews)  Type of projects analysed  16 research grants out of 556 possible grants awarded by ARC (1990-1994) | Knowledge production  302 peer-reviewed publications cited for a total of 975 times a year (between the beginning of the case studies and 2002)  Research targeting and capacity building  -24 post graduates, PhD or MD  -transfer of technological know-how and development of new lines of research  Informing policy and product development  -4 citations in SRs  -7 citations in guidelines or technology appraisals  -industrial research stimulation  -indirect support to the development of a class of drugs used outside the arthritis field  Health and health sector benefits  -reduction of the risk of recurrent  miscarriages  -improvement in symptom relief and physical functioning  - identification of other risk factors  Broader economic benefits  unquantified returns in the  reduction of days off work and the value of production gained  from having a more healthy workforce. | The payback framework is feasible to prospectively monitor the returns of  research works.  All types of grants seem to produce a range of research outputs and outcomes beyond the  usually assessed publications in the  peer-reviewed literature. |
| Grant 2000 [24] | To develop a methodology for evaluating non­commercial biomedical and health  services research | UK | Not reported | Mixed  (Public funder: NHS, Medical Research Council and charity, Wellcome Trust) | Methodological approach:  Bibliometric and citational on primary-publications in the peer –reviewed journals  and secondary- evidence based clinical guidelines outputs  Data source:  15 UK clinical guidelines | Median age of cited paper: 8 years  Research paper journal citation: 2043/2501  Authors’ country  USA: 36%  UK: 25%    Type of publication  75% research paper (mainly clinical observation) | Analysis of the evidence base of clinical  guidelines may be one way of tracking intermediate outputs of research |
| Ottoson 2009, Gutman 2009 [33,34] | To evaluate the research program Active Living Research (ALR)  aimed at increasing physical activity in the population | USA | 2001-2007 | Charity Organization: Robert Wood Johnson Foundation (RWJF) | Methodological approach: Logic model  Qualitative methods:  Key-informant interviews (ALR grantees; funding organizations other  than the RWJF; policy and advocacy organizations; ALR leadership, RWJF staff and advisors)  Quantitative methods:  ALR-supported investigator survey, grant abstract analysis, conferences and seminars evaluation | 83 projects evaluated (only 16% completed at the time of the evaluation)  Building the knowledge base:  - new research field;  -development of a strategic agenda;  -recognition of physical activity as part of energy-balance equation;  -improving methodology  -40% of the funded researchers have produced at least one scientific publication.  Building human resources:  -development of multidisciplinary field and collaboration;  -23% grants funded doctoral candidates;  -63% of the interviewed grantees indicated career promotions.  Growing financial resources:  -research grants leveraged by individual investigators due (at least in part) to their ALR grant (37%);  -support from other funding organization;    Contribution to policy debate and change:  -creation of an interface between ALR and policy environment  -development of documents for policy audience (55%) and media.-related materials (49%);  -establish relationships with policy and advocacy organizations;  -increasing policy informant perceptions regarding the usefulness and relevance of ALR  25% of the grantee investigators stated that their ALR research had had a policy impact (non federal and federal levels). | The ALR program addressed its mission to develop a knowledge base on policy and environmental factors conducive to physical activity in daily life and to create a trans disciplinary field of research. |
| Kalucy 2009 and Kalucy 2007 [27,28] | to determine the impact of  a sample of competitively funded, primary health care research projects | Australia | 2006-2008 | Public funder: Australian Research Quality Framework | Methodological approach: modified payback framework  Bibliometric analysis, desk analysis, web-based questionnaire and telephone interviews with leaders of research teams and nominated users of research.  Type of projects analysed:  four (phase I) and 17 (phase II) Australian primary health care research projects  funded by national competitivegrants. | Phase I: Bibliometric and citational analysis:  -8 publications,  -2 citations in SRs.  -many examples of dissemination material  No reference to use of results of the considered projects in documents of a political nature.  Comprehensive impact in creating a link between researchers and primary care doctors and policy makers  Improvement in organisation of services at a local and systematic level  Impact on career development, further funding and collaboration.  Phase II: web-based questionnaire Application of findings to decision making (four projects);  Contribution to improvements in service delivery at system or local level (seven projects);  Awarding of eleven PhDs, further research funding and collaborations;  39 peer reviewed papers in 26 different journals and multiple other outputs. | The intended impacts were most often achieved in areas under the control or influence of the researcher, such as researcher development, knowledge production and ongoing research. and employment. Bibliometric analysis was of little relevance in this sample. Desk analysis  provided evidence of dissemination rather  than of impact.  The payback framework and logic model were a sound basis for assessing impact. |
| Stryer 2000 [29] | To assess the outputs of  Outcome and Effectiveness Research (OER) funded by the Agency for Healthcare Research and Quality (AHRQ) | USA | 1989-1997 | Public funder: AHQR | Methodological approach: AHQR-developed framework  Interviews to principal investigators | Response rate : 61/91 (64%)  Main impact categories (salient findings):  -Descriptive epidemiology: incidence, prevalence, risk factors;  -Comparative effectiveness: outcome comparison (“what works best”);  -Economic assessment: cost benefit, cost of disease;  -patient-reported outcomes: quality of life (QoL)  - practice variations, prescriptive  - sociology of health care  -methodological development  -modelling: decision analysis, cost efficacy models  -quality of care  - SRs/meta-analysis  -legislative and regulatory actions | Descriptive epidemiology, comparative effectiveness, and economic assessment were the most cited categories of impact. A framework for assessing impact based on 4 levels, was developed:  -research finding;  -impact on policies;  -impact on clinical practice;  -impact on health care outcomes |
| Kwan 2007[32] | To evaluate outcomes and explored factors associated with the translation of research findings to changes in  health policy | Hong Kong | 1999-2006 | Health and Health Service Research Fund (HHSRF) | Methodological approach: payback framework  Questionnaires to the principal investigators of the completed research projects supported | Response rate : 87% (178/205)  Knowledge production:  -154/178 (86.5%) of the projects reported publications (average 5.4 publications per project);  -377 peer reviewed (of which 295, 78.2% have an average of 1.9 citations/year).  Research targeting and capacity building:  - 61/178 (34.3%) of the projects reported career advancements;  -80/178 (45%) of the projects reported the development of new research line.  Informing policy, behaviour change, health service benefits:  -35.4% of the projects reported impact  on informing policy through guidelines, protocols, reference standards, and Cochrane reviews;  -participation of PIs in health-related  policy/advisory committees.  -49.4% of the projects reported changes in behaviour and clinical practice. | The HHSRF has resulted in substantial outcomes as measured by a multi-level payback framework. Recall bias and measurement errors were the main limitation of the study |
| Johnston 2006 [31] | To estimate the public return on investment of clinical trials | USA | 1977- 2000 | Public funder: US National Institute of Neurological Disorders and Stroke  Prospective: society as a whole (discount 3-5%) | Methodological approach: econometric  SRs and cost-effectiveness analysis  Bibliometric analysis with detail on the publications of the cost-effectiveness evaluations  Type of projects analysed: phase III randomised trials funded by the US National Institute of Neurological Disorders and Stroke  Outcomes:  quality adjusted years of life (QALY), costs, returns and benefits evaluated at 10 years | 28 RCTs funded before 2000 for a total cost of US$ 335 million;  6/28 (21%) RCTs have improved the state of health  4/28 (14%) RCTs have contributed in making savings for the society  4/28 (14%) RCTs have increased costs for the society  QALY: 470,000 at an additional cost of US$ 3.3 billion  Net cost per QALY: US$ 7700  Overall incremental net benefit: US$ 15.2 billions  Return on investments : 4600% | The public return on investment in clinical trials has been substantial. Although the trials have  led to increased expenditures on health, the resultant health benefits have a much greater value than these  costs, even when valued conservatively, with an overall net benefit of the program of $15·2 billion at 10 years. |
| Shah 2001 [30] | To evaluate the projects funded in 1993 by the Public Health Research and Development Committee (PHRDC) | Australia | 1993 | Public funder: National Health and Medical Research Council (NHMRC) | Methodological approach:  NHMRC Research Outcomes Evaluation Model (evaluation of publications, training projects, non-NHMRC funding, dissemination activity, influence on policy-making and on clinical practice)  Survey to all chief investigators who had been awarded new project grants (n=32) and those receiving continuing project grants (n=31). | Response rate: 38/55 (69%)  -30/38 (79%) of the projects reported publications;  -total number of publications: 218 (of which 126 peer reviewed);  -23/38 (61%) projects have led to academic qualifications (25 PhD, 14 MPH, 8 MSc, 2 MMed, 1 MClinEpi, 1 MD);  -22/38 (58%) project have influenced policy making and 26/38 (69%) clinical practice. | A large proportion of  projects funded by PHRDC generated peer-reviewed publications and  provided research training.  Recipients perceive their research has influenced  policy and practice. |
| Ruch-Ross 2008 [35] | To examine the capacity of community-based health programs to conduct project evaluations and determine the impact of project evaluation on project outcome. | USA | 1989-2003 | Public funder: “Healthy Tomorrow Partnership for Children Program (HTPCP)” (Federal funding in collaboration with the American Pediatric Association) | Methodological approach:  not specified (analysis of factors other than financial support that make a difference for community-based programs of capacity to document outcomes)  Structured discussions with HTPCP project directors and staff  Survey to project  directors of HTPCP  149 community-based programs on child healthcare | Response rate (after 3 reminders via mail): 126/149 (85%).  50% of the project directors interviewed reported to have adequately evaluated the projects.  102/126 (81%) projects have produced useful information to :  -improve services (69%)  -sponsor interventions on a level of the population (58%)  -obtain new funding (51%)  -extend projects (35%)  -promote modifications in policy making (24%)  62% of the projects reported both process and outcomes indicators.  Differences in the proposed indicators (change in knowledge, in behaviour, reduction in number of prevalence and incidence of emergencies) emerged.  The projects reporting at least one outcome are more often associated with a positive conclusion. | Evaluation of community-based programs, although challenging, is beneficial to project success and  sustainability. The community partnerships should be encouraged to  incorporate evaluation into their planning process. |
| Australian Society for Medical Research 2008 [9] | To assess the historical returns on  investment to health research and development in Australia | Australia | 1960-2007 | Public funder:  Australian Society for Medical Research | Methodological approach: econometric  Cost-benefit analysis (case-studies):  estimation of the life expectancy and quality of life gains experienced by Australians over the 40-year period, in terms of reductions in disability  adjusted life years (DALYs),and placed a dollar value on these gains using the concept of the value of a statistical life (VSL) from the willingness to pay (WTP) literature  Estimation of net benefit of Australian research  All the estimations are referred to 2023. | DALYs prevented (relative to  1993 burden of disease levels):  1.34 million (each 1000 inhabitants)  Applying the VSLY to the total number of DALYs averted, the annual value of gains in wellbeing from health R&D expected to result from all impacts on health (not just Australian R&D) are over $100 billion for females and over $270 billion for males by 2023.  Estimate in net benefit of Australian research :  29.5 billion Australian dollars (2.3 billion a year)  Return on investment : 117%  Ratio cost/benefit: 2.17 (90% CI 1.16-3.34, min 0.57-6.01) | For every dollar invested in biomedical research in Australia the returns are 2.17 dollars in terms of benefits in health.  A slight reduction is observed in respect to the 2003 analysis (Ratio cost/benefit: 2.4 because of an increase in the expenses and an increase in the impact of chronic illness due to a longer life span.  Investment in research has excellent economic returns. |
| Cutler 1999 [36] | To evaluate the economics of better health, and in particular the factors in reduced  cardiovascular disease mortality.. | USA | Not reported | Public funder: National Bureau of Economic Research | Theoretical approach based on 5 potential factors responsible for better health:   1. economic growth (e.g. better nutrition) 2. new knowledge 3. medical technologies 4. better information and access (to cures) 5. law and regulatory actions   Methodology: reviews of literature, experts in the field, analysis of guidelines , data from insurance companies and medications prescriptions. | Three factors mainly contributed to decreased cardiovascular mortality:  -new technologies: 33%  (improvement in acute management -20%; better pharmacological treatments for hypertension and high cholesterol -13%);  -Better information on risk factors (smoking, hypertension: 65%  -Political actions (taxes on smoking): 10%  Estimate on return of expenses invested in information to obtain benefits : 350:1  Estimate on return of expenses invested in research to obtain benefits: 2.5:1 | Between 1950 and 1990 the decrease in mortality due to cardiovascular illness has increased life expectancy by 3.5 years equal to around US$ 1.5 trillion/year. A third of this increase can be attributed to biomedical research.  The investments in both improving information and in research have been fully exceeded by the obtained results. |
| Linee Guida CIVR 2006, Santori 2008 [25,26] | To evaluate the impact of a research project aimed at increasing transplants of organs (liver) | Italy | 2004-2005 | Public funder: Health Ministry | Methodological approach: CIVR model  (bibliometric analysis of the products of teams involved in the project) | Publications in journals peer reviewed:  62 (excluding letters and editorials)  Average Impact Factor  2.64±1.74 | The analysis of the number of publications and the impact factor is not sufficient to supply a complete evaluation of the project being analysed. The development of an *ad hoc* computer system and the coordination efforts have an important weight. |
