# Supplementary material for: Conceptual frameworks and empirical approaches used to assess the impact of health research: an overview of reviews
Source: Health Res Policy Syst. 2011 Jun 24;9:26. doi: 10.1186/1478-4505-9-26 (PMC3141787; doi:10.1186/1478-4505-9-26)
Supplement: Additional file 2 — Table s1. Qualitative description of the included SRs [file 1478-4505-9-26-S2.DOC]

**Additional file 1, Table S1. Search strategy used for Medline (up to May 2009) and website searched to retrieve relevant report not published in the scientific journals**

| [#19](http://www.ncbi.nlm.nih.gov/sites/?querykey=19&dbase=pubmed&tab=History&querytype=eSearch&) | Search **(#18) OR (#14)** | 10:19:57 | [**1034**](http://www.ncbi.nlm.nih.gov/sites/?cmd=HistorySearch&querykey=19&tab=&) |
| --- | --- | --- | --- |
| [#18](http://www.ncbi.nlm.nih.gov/sites/?querykey=18&dbase=pubmed&tab=History&querytype=eSearch&) | Search **((#17) OR (#16)) OR (#15)** | 10:19:38 | [279](http://www.ncbi.nlm.nih.gov/sites/?cmd=HistorySearch&querykey=18&tab=&) |
| [#17](http://www.ncbi.nlm.nih.gov/sites/?querykey=17&dbase=pubmed&tab=History&querytype=eSearch&) | Search **#13** Limits: **Publication Date from 1990, Humans, Meta-Analysis** | 10:19:04 | [5](http://www.ncbi.nlm.nih.gov/sites/?cmd=HistorySearch&querykey=17&tab=&) |
| [#16](http://www.ncbi.nlm.nih.gov/sites/?querykey=16&dbase=pubmed&tab=History&querytype=eSearch&) | Search **#13** Limits: **Publication Date from 1990, Humans, French, Italian, Spanish** | 10:17:43 | [42](http://www.ncbi.nlm.nih.gov/sites/?cmd=HistorySearch&querykey=16&tab=&) |
| [#15](http://www.ncbi.nlm.nih.gov/sites/?querykey=15&dbase=pubmed&tab=History&querytype=eSearch&) | Search **#13** Limits: **Publication Date from 1990, Humans, Review** | 10:17:17 | [239](http://www.ncbi.nlm.nih.gov/sites/?cmd=HistorySearch&querykey=15&tab=&) |
| [#14](http://www.ncbi.nlm.nih.gov/sites/?querykey=14&dbase=pubmed&tab=History&querytype=eSearch&) | Search **(#12) OR (#11)** Limits: **Publication Date from 2007 to 2009** | 10:15:49 | [828](http://www.ncbi.nlm.nih.gov/sites/?cmd=HistorySearch&querykey=14&tab=&) |
| [#13](http://www.ncbi.nlm.nih.gov/sites/?querykey=13&dbase=pubmed&tab=History&querytype=eSearch&) | Search **(#12) OR (#11)** | 10:15:33 | [3086](http://www.ncbi.nlm.nih.gov/sites/?cmd=HistorySearch&querykey=13&tab=&) |
| [#12](http://www.ncbi.nlm.nih.gov/sites/?querykey=12&dbase=pubmed&tab=History&querytype=eSearch&) | Search **PAYBACK OR “PAY BACK” OR Buxton M** | 10:15:18 | [413](http://www.ncbi.nlm.nih.gov/sites/?cmd=HistorySearch&querykey=12&tab=&) |
| [#11](http://www.ncbi.nlm.nih.gov/sites/?querykey=11&dbase=pubmed&tab=History&querytype=eSearch&) | Search **((#10) AND (#6)) AND (#5)** | 10:14:11 | [2678](http://www.ncbi.nlm.nih.gov/sites/?cmd=HistorySearch&querykey=11&tab=&) |
| [#10](http://www.ncbi.nlm.nih.gov/sites/?querykey=10&dbase=pubmed&tab=History&querytype=eSearch&) | Search **((#7) OR (#8)) OR (#9)** | 10:07:44 | [50177](http://www.ncbi.nlm.nih.gov/sites/?cmd=HistorySearch&querykey=10&tab=&) |
| [#9](http://www.ncbi.nlm.nih.gov/sites/?querykey=9&dbase=pubmed&tab=History&querytype=eSearch&) | Search **"Qualitative Research"[MAJR]** | 10:07:27 | [800](http://www.ncbi.nlm.nih.gov/sites/?cmd=HistorySearch&querykey=9&tab=&) |
| [#8](http://www.ncbi.nlm.nih.gov/sites/?querykey=8&dbase=pubmed&tab=History&querytype=eSearch&) | Search **(implement* OR disseminate*) AND (benefit* OR impact* OR gain*)** | 10:07:15 | [28816](http://www.ncbi.nlm.nih.gov/sites/?cmd=HistorySearch&querykey=8&tab=&) |
| [#7](http://www.ncbi.nlm.nih.gov/sites/?querykey=7&dbase=pubmed&tab=History&querytype=eSearch&) | Search **"Diffusion of Innovation"[Mesh] OR "Investments"[Mesh] OR "Financing, Organized"[Mesh:NoExp]** | 10:07:05 | [21093](http://www.ncbi.nlm.nih.gov/sites/?cmd=HistorySearch&querykey=7&tab=&) |
| [#6](http://www.ncbi.nlm.nih.gov/sites/?querykey=6&dbase=pubmed&tab=History&querytype=eSearch&) | Search **"Data Collection"[Mesh] OR "Bibliometrics"[Mesh]** | 10:06:26 | [1026942](http://www.ncbi.nlm.nih.gov/sites/?cmd=HistorySearch&querykey=6&tab=&) |
| [#5](http://www.ncbi.nlm.nih.gov/sites/?querykey=5&dbase=pubmed&tab=History&querytype=eSearch&) | Search **"Health Policy"[Mesh] OR "Health Services Research"[Mesh] OR "Program Development"[Mesh] OR "Program Evaluation"[Mesh] OR "Nursing Evaluation Research"[Mesh] OR "Technology Assessment, Biomedical"[Mesh] OR "Health technology assessment" OR HTA** | 10:05:08 | [193041](http://www.ncbi.nlm.nih.gov/sites/?cmd=HistorySearch&querykey=5&tab=&) |

| **Istitutions/Foundations/University** | **Country** | **URL** |
| --- | --- | --- |
| Brunel University | UK | [www.brunel.ac.uk](http://www.brunel.ac.uk/) |
| Medical Research Council | UK | [www.mrc.ac.uk/](http://www.mrc.ac.uk/) |
| Research Councils UK | UK | [www.rcuk.ac.uk/](http://www.rcuk.ac.uk/) |
| UK Clinical Research Collaboration | UK | [www.ukcrc.org](http://www.ukcrc.org/) |
| Research Assesment Exercise | UK | [www.rae.ac.uk/](http://www.rae.ac.uk/) |
| Health Technology Assessment programme | UK | [www.hta.ac.uk/](http://www.hta.ac.uk/) |
| Wellcome Trust | UK | [www.wellcome.ac.uk](http://www.wellcome.ac.uk/) |
| Australian Society of Medical Research | Australia | [www.asmr.org.au](http://www.asmr.org.au/) |
| Primary Health Care  Research & Information Service | Australia | [www.phcris.org.au/](http://www.phcris.org.au/) |
| Canadian Academy of Science | Canada | [www.cahs-acss.ca](http://www.cahs-acss.ca/) |
| Canadian Health Services Research Foundation | Canada | [www.chsrf.ca](http://www.chsrf.ca/) |
| Council for International Organizations of Medical Sciences | International | [www.cioms.ch/](http://www.cioms.ch/) |
| The Robert Wood Johnson Foundation | USA | [www.rwjf.org/](http://www.rwjf.org/) |
| The Rand Corporation | USA | [www.rand.org](http://www.rand.org/) |
| The Lasker Foundation | USA | [www.laskerfoundation.org/](http://www.laskerfoundation.org/) |
| Institute of Medicine of National Academy | USA | [www.iom.edu](http://www.iom.edu/) |
| National Institute of Health | USA | [www.nih.gov/](http://www.nih.gov/) |
| The Bill & Melinda Gates Foundation | USA | [www.gatesfoundation.org](http://www.gatesfoundation.org/) |
| Swedish Research Council | Sweden | [www.vr.se](http://www.vr.se/) |
| Royal Netherlands Academy of Arts and Sciences | The Netherlands | [www.knaw.nl/](http://www.knaw.nl/) |
| Ministero della Salute | Italia | [www.salute.gov.it/](http://www.salute.gov.it/) |
| Ministero dell’Istruzione, Università e Ricerca | Italia | [www.miur.it](http://www.miur.it/) |
| Comitato di Indirizzo per la Valutazione della Ricerca | Italia | <https://civr.cineca.it/> |
| Telethon Italia | Italia | [www.telethon.it](http://www.telethon.it/) |
