# Supplementary material for: Conceptual frameworks and empirical approaches used to assess the impact of health research: an overview of reviews
Source: Health Res Policy Syst. 2011 Jun 24;9:26. doi: 10.1186/1478-4505-9-26 (PMC3141787; doi:10.1186/1478-4505-9-26)
Supplement: Additional file 3 — Table s2. Qualitative description of primary studies not included in the previous mentioned reviews [file 1478-4505-9-26-S3.DOC]

**Additional file 2, Table S2. Qualitative description of the included SRs**

| **Bibliographic reference** | **Aim and Focus** | **Country** | **Commissioner** | **Evaluation time frame** | **Methods** | **Main Findings** | **Main conclusions** |
| --- | --- | --- | --- | --- | --- | --- | --- |
| Hanney 2007 [12] | to consider how the impact of the National Health Service (NHS)  Health Technology Assessment (HTA) program should be measured | UK | Public funder: NHS | 1990-2005 | literature review of research program, review of the work of the National  Coordinating Centre for Health Technology Assessment (NCCHTA), survey of the lead researchers | 46 studies included:  -5 conceptual or methodological  approaches;  -23 empirical applications or evaluations of the impact of a particular program of health  -18 joint presentation of  conceptual approaches and their application  Methods used:  -document evaluation (administrative, bibliometric)  -Questionnaires  - Interviews  -Case studies | A large diversity in the existing approaches to research impact evaluation emerged.  The frameworks identified tended  to be focused on specific impact assessment initiatives for designated research programs or groups  The majority of the described models  adopt multidimensional evaluations.  More than 50% of the studies make reference to the payback model.  The case study seems to represent the stronger methodology.  The main difficulties were linked to the assessment of the later categories of impact. |
| Frank 2009, Canadian Academy of Health Science 2009 [19,20] | To establish a Canadian model to evaluate the impacts of health research and to define the “best metrics” that could be used to assess those impacts (or improve them) | Canada and the rest of the world | Public funder:  federal, provincial, university | Not reported | SR and international panel activity  Analysis of  Canadian Experiences (N=6):  Regional and national  International Experiences (N=6):  -USA  -UK  -Netherlands  -Sweden  -Australia  -Other countries | 5 macro areas where research impact is measured:  -Advancing  knowledge  -Research capacity‐building  -Informing decision‐making  -Health‐impact  Broad  economic  and  social  impacts  For the 5 identified macro areas specific indicators are suggested. | No single model fully captures the global impact of biomedical research.  From the analysis the existing approaches, sets of indicators have been identified and their potential application defined  Characteristics of an optimal indicator:  -Convenience: validity, behavioural impact, simplicity, coverage, appropriate time window, soundness, replicability, comparability, relevance, responsiveness.  -Feasibility: easy and cheap to be collected, data availability, compliance cost, transparency, timeliness, attribution, avoid gamesmanship, interpretation, well-defined.  Many aspects cannot be generalized and strictly depend on the type of funding, the needs of the stakeholders involved and by costs. |
| Buxton 2004 [18] | To assess what can be learnt from previous studies that have estimated monetary values for the societal  benefits obtained from health research | Global | WHO | Not reported | SR of studies that have attempted to link (and value) benefits to a specific  society from a specified (and costed) body of research.  Benefits were classified in:   - Direct costs saved for health care systems - indirect costs saved ( eg healthier human capital) - commercial development - intrinsic value for society linked to improvement in state of health | 31 publications included, mainly coming from industrialized countries (USA)  Direct costs:   - new therapies reducing the number of patients having to be treated or costs per patient (e.g. vaccines)   Indirect costs:   - Improvement in productivity due to a reduction in morbidity and mortality coming from biomedical research.   Commercial development:   - Growth in employment, start up of new industries, industrial growth   Intrinsic value for society:   - Economic value in the increase of longevity | A growing evidence body demonstrates  that health and biomedical research is an investment: there are tangible benefits and it is quite possible that exceptionally attractive long-term returns may accrue. Substantial efforts are needed to refine existing methodologies and to make them more robust.  A fundamental problem is that counterfactual cannot be observed (what would have happened if the research in question had not been funded). |
| Coryn 2007 [21] | To describe, classify, and comparatively evaluate national models and  mechanisms used to assess research and allocate research funding in 16 countries | Global | Public funder: government | Not reported | Review of detailed country narratives  rated by independent, blinded panels of professional researchers and evaluators in two countries. Evaluation was based on 25 quality indicators (validity, credibility, utility, cost-effectiveness, and ethicality) | The analysed models can be classified in:  Type I: Large-scale performance/judgment-based exercises of various types and classes (i.e., performance-based funding, United Kingdom’s  Research Assessment Exercise (RAE))  Type II: Bulk funding models (e.g., block grants to large groups, Japanese research system)  Type III: Indicator-driven models (e.g., funding based on algorithms using student numbers in research programs or institutions, bibliometric indicators, Australia’s Relative Funding Model (RFM)). | The New Zealand’s Performance-Based Research Fund (PBRF) is considered the highest quality model for evaluating research and allocating research funding in comparison to the other 15 national models included in the sample  Overall, the highest ratings were received by nations using large-scale research assessment exercises. Bulk funding and indicator-driven models received substantially lower ratings. |
